# Exploring family educational involvement and social skills in Chinese preschoolers: The moderating role of parent-child relationship

**DOI:** 10.3389/fpsyg.2022.911421

**Published:** 2022-08-01

**Authors:** Hao Liu, Yuxi Qiu, Li Luo

**Affiliations:** ^1^College of Preschool Education, Capital Normal University, Beijing, China; ^2^College of Arts, Sciences and Education, Florida International University, Miami, FL, United States

**Keywords:** home-based involvement, parent-child closeness, social skills, preschool children, China

## Abstract

The purpose of this study was to examine parent-child relationship as a moderator of the association between family educational involvement and the social skills of preschoolers. A total of 4,938 children (*M* = 5.09-years-old, *SD* = 0.81) were sampled from 18 preschools in Hebei province, China, and their parents completed a survey packet to collect demographic information, as well as ratings of parental involvement, relationships with their children, and child social skill development. The results of multivariate regression analysis suggested that: (1) both home-based involvement and home-school conferencing could significantly predict preschoolers’ social skills, (2) there was stronger evidence for a relationship of home-based involvement and preschoolers’ social skills, (3) closeness in parent-child relationship moderated the path from home-based involvement to preschoolers’ social skills, and (4) there was no interactive effect between family educational involvement and parent-child conflict. These findings highlight the significance of the joint influences of family educational involvement and parent-child relationship in shaping children’s social skills. The impact of home-based involvement was boosted in the context of a close parent-child relationship.

## Introduction

A considerable body of research has established a positive relationship between the social skills of preschoolers and their readiness for school (e.g., McClelland et al., 2000; Rhoades et al., 2011). Parents are considered an important source of support for the development of child social skills and the significant role of family educational involvement in fostering children’s social skills is well documented (e.g., McWayne et al., 2004; Brajša-Žganec et al., 2019). Family educational involvement is characterized by both micro- and mesosystems, which not only serve as an immediate environment, but also bridge the home and school settings during child development (Seginer, 2006; El Nokali et al., 2010).

According to Darling and Steinberg’s (1993) Integrative Model of Parenting, relationships between parents and children cultivate the emotional context for unfolding parenting practices, including their involvement in children’s development and learning; however, few empirical studies have been conducted to examine the moderating effect of parent-child relationship. Furthermore, previous research has been heavily focused on school-age children and within the context of Western culture (Fan and Chen, 2001; See and Gorard, 2015). To consider the associations between family educational involvement, parent-child relationship, and preschoolers’ social skills, in the present research we investigated whether parent-child closeness and conflict moderated the potential relationship between family educational involvement and preschoolers’ social skills in the Chinese context.

### Association between family educational involvement and children’s social skills

As a multifaceted construct, family involvement refers to a variety of parental behaviors in the home, school, and community environments, that promote positive educational outcomes for their children (Seginer, 2006). Focusing on the home-school partnership, Epstein (1995) developed a framework to illustrate six types of family involvement, including parenting, participating in communication with schools, volunteering in school activities, providing learning support at home, getting involved in school decision-making processes, and engaging with community resources. In consideration of developmentally appropriate family involvement for young children, Fantuzzo et al. (2000) categorized it into three types: school-based involvement, home-school conferencing, and home-based involvement. As Chinese traditional culture places a great emphasis on the responsibility of parents for their children’s education, Chinese parents generally exhibit high levels of parental involvement (Cheung and Pomerantz, 2011); however, previous research has suggested that Chinese parents of young children were less involved in school than at home (Lau et al., 2011; Xia et al., 2020), probably due to respect for and deference to the authority of teachers (Pang, 2004).

Although previous studies on the impact of family educational involvement have primarily focused on academic performance, a growing literature has documented positive relationships between family involvement and social functioning (e.g., Torres et al., 2014; Cohen and Anders, 2020). For example, using data from the NICHD Study of Early Childcare and Youth Development, El Nokali et al. (2010) found that children with highly involved parents showed better social skills and fewer problem behaviors, and that increases in parent involvement longitudinally predicted rapid growth in social skills and decreases in problem behaviors. Furthermore, dimensions of family involvement were found to be differently associated with child outcomes. In a sample of 103 kindergarten children and their mothers, Hill and Craft (2003) found parental involvement at home was positively linked to children’s prosocial communication and emotional regulation. Researchers further suggested the home-based parental involvement had a greater impact on young children’s self-control, cooperative behaviors, and social engagement, when compared with other family involvement dimensions (Fantuzzo et al., 2004; McWayne et al., 2004). The relationships between school-based involvement, home-school conferencing, and child outcomes are mixed. Some researchers found parental involvement at school positively predicted young children’s social skill (Powell et al., 2010) and school adjustment (Kang et al., 2017). It has been suggested that school-based involvement and home-school conferencing increase parents’ awareness of their child’s social and behavioral difficulties at school, make them better prepared to address these issues at home, and have them be more informed of school and community resources that could benefit their child both socially and academically (Hill and Taylor, 2004; Lau et al., 2011). However, several other studies found no or even negative relationships. For example, Rimm-Kaufman et al. (2003) found family involvement activities at school were not associated with seven of the nine social and academic outcomes, and more school-based involvement was linked to higher behavior problems in kindergarten children. When children exhibit more behavior problems at school, parents are more likely to get involved and communicate with teachers.

### Parent-child relationship as a moderator

The relationship between parents and children during the early years is recognized as a critical protective factor for children’s developmental outcomes. High-quality parent-child relationships provide children with a context where they can learn and develop social skills. Substantial studies have documented that children with better relationships with parents exhibited higher levels of social and academic development than their counterparts with lower-quality relationships (e.g., Connell and Prinz, 2002). Specifically, parental warmth and expressivity (Zhou et al., 2002), emotional support (Denham et al., 1997), and closeness in parent-child relationship (Leidy et al., 2010) are significantly and positively linked to preschoolers’ social skills. A close relationship with parents in the early years fosters a secure attachment and forms the foundation of the child’s future social interactions. In a warm and positive relationship atmosphere, parents openly and effectively communicate with their child, become sensitive to their child’s needs and cues, and show emotional availability toward the child (Edwards et al., 2010). When having a close parent-child relationship, children tend to learn how to self-regulate through observing and mimicking their parents, and practice friendship skills with their parents. However, research on conflict in parent-child relationships has yielded inconsistent results. There have been reports that parent-child conflict is associated with problem behavior of children, peer rejection, and school adjustment (Weaver et al., 2015; Xu et al., 2018), while other researchers support the benefits of parent-child conflict in promoting social skills (Laible and Thompson, 2002; Boyer et al., 2016). For example, researchers suggest that family conflict experiences can provide children with potential learning opportunities to discuss social rules and behavioral expectations (Zhang, 2013), regulate negative emotions such as anger, sadness, and hurt (Denham, 2007), and engage in social problem solving with their parents (Nelson, 2015), which serve as a basis for their development of social skills.

Based on the Integrative Model of Parenting (Darling and Steinberg, 1993), the overarching emotional climate of the parent-child relationship is considered a contextual variable that moderates associations between parenting practices (including parental involvement) and child development. Parent-child relationship provides an emotional context in which children are experiencing parental involvement in their education. When parental involvement is characterized by emotional support and positive affect, it can be beneficial to children; but when it is marked by control, negative affect, and intrusiveness, it can be detrimental to children (Pomerantz et al., 2007). In the context of high parent-child closeness, children tend to perceive their parents’ educational involvement as an expression of love and support, and therefore, are more motivated to perform better; however, in the context of high parent-child conflicts, children might perceive their parent’s educational involvement as an intrusion into their daily lives, and are less motivated to well behave (Stright and Yeo, 2014). Within a close atmosphere, children are more open to their parents’ socialization practices (Spera, 2005). Increases in parent-child closeness may support children’s internalization of parental beliefs and behaviors, and make them more likely to engage in expected activities/interactions, which would, in turn, lead to children’s higher achievement. In contrast, when parents are perceived as more demanding, controlling, and negative, children are more likely to be resistant to their parents’ educational involvement; and thus the benefits of parents’ educational involvement may be weakened (Xia et al., 2020). In a sample of 175 kindergartners in the United States and their mothers, Simpkins et al. (2006) found that the associations of family educational involvement to children’s academic outcomes differed depending on the mother-child relationship. Specifically, as maternal perceptions of mother-child warmth increased, the favorable relationship between family involvement and children’s mathematics and literacy achievement became stronger. Focused on elementary students and their parents in Singapore, Stright and Yeo (2014) also found that the warmth of parent-child relationship enhanced the linkage between parental school-based involvement and their children’s academic achievement. However, empirical evidence on the moderating role of parent-child relationship on children’s social skills is rare. In addition, further studies are needed to better understand the context in which family educational involvement predicts children’s social skills (Xia et al., 2020).

### The present study

**FIGURE 1:**
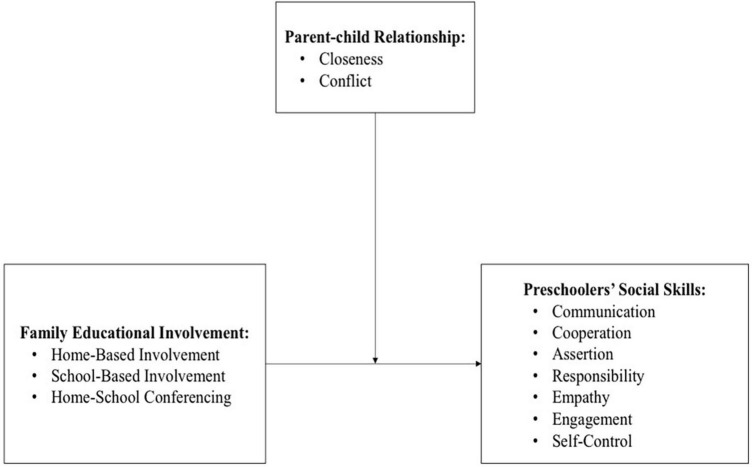
Conceptual model of the relation between family educational involvement, parent-child relationship, and social skills among preschoolers.

Given the strong links between both family educational involvement and parent-child relationship and social skills of young children, it’s critical to explore the relationship between these two factors, especially in the context of how these variables interact. The purpose of this study was to examine the moderating effect of parent-child relationship in the association between family educational involvement and their children’s social skills. Informed by previous studies showing that child development outcomes are a product of the interplay between family educational involvement and parent-child relationship (e.g., Darling and Steinberg, 1993; Simpkins et al., 2006; Stright and Yeo, 2014), we proposed that parent-child relationship emerged as a moderator in this study (see Figure 1). Specifically, the present study focused on the following two research questions: (1) To what extent is family educational involvement associated with preschoolers’ social skills? and (2) Does parent-child relationship (closeness or conflict) moderate the relationship between family educational involvement and preschoolers’ social skills?

## Materials and methods

### Participants

Study participants were from Hebei province, which is located on the northern coast of China. With a population of more than 74 million, the gross domestic product of Hebei province ranked 12 out of 31 mainland Chinese provincial-level administrative regions in 2020 (National Bureau of Statistics of China, 2021). We used stratified sampling to select study participants. Three cities in Hebei province were purposively selected to represent low, middle, and high levels of economic development, which were classified by the Hebei provincial government. Then, in each of the three cities, we selected 5–6 preschools, giving a total of 16 preschools. Finally, from each sampled preschool, nine classes were selected, with three for each age group: 3–4, 4–5, and 5–6 years old. All parents with children enrolled in the selected classes were invited to participate in this study. A total of 5,300 survey packets were sent home to parents and 4,938 were returned, indicating a 93.2% response rate.

As shown in Table 1, the mean age of children was 5.09 years (*SD* = 0.81). The sample included 2,402 (48.6%) girls and 2,536 (51.4%) boys, and 1,139 (23.1%) were the only child in their families. Of the respondents, 4,049 (82.0%) of the questionnaires were completed by mothers, 843 (17.1%) were completed by fathers, and 46 (0.9%) were completed by other guardians. Of the fathers, 39.0% had obtained a bachelor’s or higher degree, as had 47.4% of the mothers. Further, 44.0% of fathers and 45.7% of mothers were between the ages of 31 and 35 years, and 35.5 and 42.2% of fathers and mothers, respectively, reported their occupation as professionals or officers (e.g., teachers, doctors, technicians). In terms of household income, a majority of the families (55.6%) reported a medium-level monthly income (¥6,001 to ¥15,000).

**TABLE 1:** Demographic information of the sample (*n* = 4,938).

Variables	M (*SD*) or %	
***Type of Respondent***		
Father	17.1%	
Mother	82.0%	
Other	0.9%	
***Child Characteristics***		
Age (year)	5.09 (.90)	
One-only child	23.1%	
Gender		
1. Boys	51.4%	
2. Girls	48.6%	
***Family Characteristics***		
Region		
1. Urban	68.1%	
2. Rural	31.9%	
Monthly household income (scored below from 1 to 7)		
1. Less than ¥1,500	1.1%	
2. ¥1,501 to ¥4,500	13.5%	
3. ¥4,501 to ¥6,000	17.8%	
4. ¥6,001 to ¥10,000	37.8%	
5. ¥10,001 to ¥15,000	17.8%	
6. ¥15,001 to ¥20,000	6.2%	
7. ¥20,001 or more	5.8%	
Parental education (scored below from 1 to 7)	Father	Mother
1. Less than 3 years	0.1%	0.1%
2. 4–6 years	0.7%	0.5%
3. Middle school or below	11.2%	9.7%
4. High school or vocational school degree	18.9%	16%
5. Vocational college degree	30.1%	26.3%
6. Bachelor’s degree	35.7%	43.8%
7. Master’s degree or above	3.3%	3.6%
Parental occupation (scored below from 1 to 5)	Father	Mother
1. Unemployed, non-technical worker, or farmer	6.3%	17.8%
2. Semi-technical worker or self-employed small business owner (e.g., construction worker)	23.2%	13.9%
3. Technical worker or semi-professional (e.g., driver)	22.2%	20.9%
4. Professional or officer (e.g., doctor, teacher, technician)	35.5%	42.2%
5. High-level professional or administrator (e.g., manager)	12.8%	5.2%
Parental age	Father	Mother
1. 25 years old or younger	0.4%	0.8%
2. Between 26 and 30 years old	10.4%	15.0%
3. Between 31 and 35 years old	44.0%	45.7%
4. Between 36 and 40 years old	27.1%	25.3%
5. 41 years old or older	18.0%	13.2%

### Measures

#### Family involvement questionnaire-short form

The family involvement questionnaire-short form (FIQ-SF; Fantuzzo et al., 2013) was used to measure the type and extent of participation of parents or other primary caregivers in their children’s education. The FIQ-SF consists of three subscales that assess specific involvement behaviors associated with: (1) Home-based involvement, (2) School-based involvement, and (3) Home-school conferencing. The Home-based involvement subscale characterizes family involvement in the home environment (seven items; e.g., “I bring home learning materials for my child, such as tapes, videos, and books.”); the School-based involvement subscale depicts family involvement in the school environment (seven items; e.g., “I attend parent workshops or training offered by my child’s school.”); and the Home-school conferencing subscale describes the communication between parents and teachers (seven items; e.g., “I talk to my child’s teacher about my child’s accomplishments.”). Each FIQ-SF item is rated on a four-point Likert-type scale (1 = *rarely*, 2 = *sometimes*, 3 = *often*, and 4 = *always*) and ratings are summed across items for each subscale as measures of parental involvement. Previous validation studies have demonstrated that the FIQ-SF is an adequate measure of parental involvement in both the Western (e.g., Bulotsky-Shearer et al., 2016) and Chinese (e.g., Liu and Li, 2019) contexts. In the present study, Cronbach’s alpha value was 0.966 for the Home-based involvement subscale, 0.939 for the School-based involvement subscale, and 0.915 for the Home-school conferencing subscale.

#### Child-parent relationship scale

In this study, Chinese parents were asked to report their relationship with their children using the short form of the child-parent relationship scale (CPRS; Pianta, 1992). The CPRS comprises 15 items categorized into two subscales: Closeness and Conflict. The Closeness subscale measures parents’ perception of open communication and affection with their child (seven items; e.g., “I share an affectionate, warm relationship with my child.”), whereas the Conflict subscale assesses parents’ perception of negativity and collision with their child (eight items; e.g., “My child and I always seem to be struggling with each other.”). Each item on the CPRS is rated on a five-point Likert-type scale, ranging from 1 (*definitely does not apply*) to 5 (*definitely applies*). The CPRS has been widely used across many cultures, including in China, due to its adequate psychometric properties (e.g., Zhang et al., 2008; Zhang and Chen, 2010). The Closeness and Conflict subscales had Cronbach’s alpha values of 0.840 and 0.837, respectively, in the current study.

#### Social skills improvement system-rating scales

We used the parent version of the social skills improvement system-rating scales (SSIS-RS; Gresham and Elliott, 2008) to measure preschool children’s social skills. In this study, the social skills subscale of the SSIS-RS was applied, and comprises seven dimensions: (1) Communication (seven items; e.g., “Takes turns in conversations.”), (2) Cooperation (six items; e.g., “Pays attention to your instructions.”), (3) Assertion (seven items; e.g., “Questions rules that may be unfair.”), (4) Responsibilities (six items; e.g., “Respects the property of others.”), (5) Empathy (six items; e.g., “Tries to comfort others.”), (6) Engagement (seven items; e.g., “Invites others to join in activities.”), and (7) Self-control (seven items; e.g., “Stays calm when disagreeing with others.”). Items were rated on a four-point Likert-type scale (0 = *never*, 1 = *seldom*, 2 = *often*, 3 = *almost always*), with a higher total score interpreted as better social skills. The validity and reliability of the SSIS-RS social skills subscale for Chinese parents of preschool-aged children have been supported (e.g., Wu et al., 2019). In this study, Cronbach’s alpha values ranged from 0.780 to 0.854 for the seven dimensions.

#### Demographic questionnaire

The demographic questionnaire was used to collect study participant background information, including the child’s age, sex, and sibling status. Further, family socioeconomic status (SES) was indexed according to five indicators: (1) paternal education, (2) paternal occupation, (3) maternal education, (4) maternal occupation, and (5) household income. In the current study, paternal/maternal education was divided into 7 categories ranging from 1 (*less than three years*) to 7 (*master’s degree or above*), paternal/maternal occupation contained 5 categories, with a rage from 1 (*unemployment, job-waiting, part-time job, or farmer*) to 5 (*senior management personnel and senior professional*), and household income was coded into 7 levels ranging from 1 (*less than ¥ 1500*) to 7 (*more than ¥ 20,000*). Following previous research (e.g., Lin and Bian, 1991; Hu et al., 2017), we standardized the scores of the above five indicators to z-scores and then averaged these to produce the family SES index score, where a higher index score indicates a greater family SES.

### Data analysis strategies

To address our research questions, we analyzed data in the following two steps: Step 1, confirmatory factor analysis (CFA) and Step 2, multivariate regression analysis. In Step 1, a CFA model was fitted to the item-level data for each instrument (i.e., SSIS-RS, FIQ-SF, and CPRS). A CFA model was specified according to the initial measurement structure of each instrument. Specifically, for SSIS-RS, a seven-factor model was specified, where each factor corresponded to a subscale of SSIS-RS. Similarly, a three-factor model was specified for FIQ-SF and a two-factor model for CPRS. According to Kline (2016), the aforementioned models were identified because they presented a standard structure, where there were at least two indicators (i.e., items) of each factor, with no cross-loadings or correlated residuals. To complete model identification, we fixed the factor variance at one. By the end of this step, we examined the measurement structure underlying each instrument by reviewing model fit indices and factor loadings, then factor scores for the subscales of each instrument were saved for use in subsequent multivariate regression analysis.

In Step 2, we conducted a multivariate regression analysis to examine the effects of parental involvement (three subscales) and parent-child relationship (Closeness and Conflict), as well as their interactions, on the seven aspects of preschoolers’ social skills, as measured by SSIS-RS. We treated children’s age and family SES as covariates in the model. This chosen analytical technique fits adequately to our research interest, where we focused on predicting several outcomes from the same set of predictors (Stevens, 1996) and, hence, was sufficient to answer our research questions. From this step, standardized path coefficients were reviewed. Prior to multivariate regression analysis, we fitted a structural equation model with latent variable interactions, and the model could not be estimated, due to the high computational intensity caused by the large number of latent variable interactions (three FIQ-SF subscales ×two CPRS subscales = six).

For the studied models, we reviewed the following statistical indices to assess model fit (Kline, 2016) χ^2^ (statistical non-significance suggests perfect model fit), Comparative Fit Index (CFI; CFI ≥ 0.95), Tucker-Lewis Index (TLI; TLI ≥ 0.95), Root Mean Square Error of Approximation (RMSEA; RMSEA ≤ 0.08), and the Standardized Root Mean Square Residual (SRMR; SRMR ≤ 0.08), where the χ^2^ test is a test of exact model fit and the others are indicators of approximate fit. Correspondingly, the evaluation of model fit primarily relied on approximate fit indices. Data analysis was conducted using Mplus 8.4 (Muthén and Muthén, 1998-2017). As the children were nested within classrooms, we further specified CLUSTER = CLASSID and TYPE = COMPLEX, to account for the nesting structure of child data within classrooms, as well as the non-independence therein. Diagonally weighted least square (WLSMV) and robust maximum likelihood (MLR) were used for CFA and the multivariate regression analysis, respectively.

## Results

### Evaluation of measurement structure

Results from CFA are presented in Supplementary Tables 1–4. Overall, acceptable fit was found for all tested CFA models. The values of model fit indices for measures of social skills (SSIS-RS) and parent-child relationship (CPRS) were well within the range of adequate fit (RMSEA = 0.052, 90% *CI* [0.051, 0.053], CFI = 0.95, TLI = 0.95, SRMR = 0.05, for SSIS-RS; RMSEA = 0.079, 90% *CI* [0.076, 0.081], CFI = 0.96, TLI = 0.95, SRMR = 0.06, for CPRS). For the measure of parental involvement (FIQ-SF), values of CFI/TLI and SRMR fell within the acceptable range (CFI = 0.99, TLI = 0.98, SRMR = 0.04), but RMSEA slightly exceeded the cutoff (RMSEA = 0.096, 90% *CI* [0.095, 0.098]). The disagreement between RMSEA and CFI, as noted in Lai and Green (2016), does not necessarily suggest a model fits data poorly or model misspecification. Hence, in addition to the reported global fit indices, we further reviewed correlation residuals (differences between observed and model implied correlations) for understanding of local fit. Accordingly, the absolute values of the correlation residuals varied from 0.00 to 0.14, indicating the three-factor model of FIQ-SF was able to closely reproduce the observed correlation matrix. Also, considering the latent construct of FIQ-SF as supported in existing literature (e.g., Fantuzzo et al., 2013; McWayne et al., 2015; Bulotsky-Shearer et al., 2016) and the high factor loadings of items on the designated dimensions (Supplementary Table 3), we decided to retain this three-factor solution.

### Descriptive statistics and correlations

Means and standard deviations of the key study variables, as well as bivariate correlations, are presented in Table 2. Mean family educational involvement was at moderate levels across all three dimension subscales: Home-school conferencing, *M* = 2.34 (*SD* = 0.79); School-based involvement, *M* = 2.25 (*SD* = 0.82), and Home-based involvement, *M* = 2.62 (*SD* = 0.66). Mean preschoolers’ social skills scores lay between moderate and upper levels, including: Communication, *M* = 2.05 (*SD* = 0.40); Cooperation, *M* = 2.13 (*SD* = 0.40); Assertion, *M* = 1.94 (*SD* = 0.43); Responsibility, *M* = 1.98 (*SD* = 0.44); Empathy, *M* = 1.99 (*SD* = 0.43); Engagement, *M* = 2.09 (*SD* = 0.44); and Self-control, *M* = 1.66 (*SD* = 0.44). Regarding parent-child relationship, perceptions of parent-child Closeness (*M* = 4.51, *SD* = 0.50) scored higher than those of parent-child Conflict (*M* = 2.44, *SD* = 0.77).

**TABLE 2:** Bivariate correlation, mean, and standard deviation of studied variables.

	1	2	3	4	5	6	7	8	9	10	11	12	13	14
1. Communication	1.00													
2. Cooperation	0.94**	1.00												
3. Assertion	0.96**	0.83**	1.00											
4. Responsibility	0.94**	0.98**	0.85**	1.00										
5. Empathy	0.98**	0.92**	0.94**	0.93**	1.00									
6. Engagement	0.94**	0.83**	0.92**	0.81**	0.93**	1.00								
7. Self-Control	0.90**	0.89**	0.87**	0.94**	0.91**	0.79**	1.00							
8. Family SES	0.10**	0.08**	0.11**	0.08**	0.10**	0.08**	0.09**	1.00						
9. Child age	0.07**	0.09**	0.05**	0.10**	0.07**	0.07**	0.09**	−0.10**	1.00					
10. HSC	0.42**	0.38**	0.42**	0.39**	0.41**	0.40**	0.40**	0.09**	0.01	1.00				
11. SBI	0.42**	0.39**	0.42**	0.40**	0.42**	0.41**	0.40**	0.10**	0.06**	0.83**	1.00			
12. HBI	0.53**	0.50**	0.52**	0.50**	0.52**	0.51**	0.50**	0.21**	0.01	0.70**	0.75**	1.00		
13. Closeness	0.42**	0.41**	0.39**	0.40**	0.40**	0.39**	0.35**	0.09**	–0.02	0.22**	0.23**	0.35**	1.00	
14. Conflict	−0.24**	−0.27**	−0.18**	−0.25**	−0.22**	−0.20**	−0.20**	−0.10**	0.00	–0.01	–0.02	−0.11**	−0.55**	1.00
Mean	2.05	2.13	1.94	1.98	1.99	2.09	1.66	0.00	5.09	2.34	2.25	2.62	4.51	2.44
*SD*	0.40	0.40	0.43	0.44	0.43	0.44	0.44	0.53	0.81	0.79	0.82	0.66	0.50	0.77

HSC, home-school conferencing; SBI, school-based involvement; HBI, home-based involvement.

^**^ 0.01.

As shown in Table 2, significant positive correlations were found between preschoolers’ social skills in the seven subscale aspects and Home-school conferencing, School-based involvement, and Home-based involvement (0.53 ≥ *r* ≥ 0.38; *p* < 0.01). Closeness in parent-child relationship was positively correlated with preschoolers’ social skills (0.42 ≥ *r* ≥ 0.35; *p* < 0.01), whereas negative correlations were observed with Conflict in parent-child relationship (−0.18 ≥ *r* ≥ –0.27; *p* < 0.01). Closeness in parent-child relationship and scores for each of the family educational involvement subscales were positively correlated (*r*, 0.22–0.35). Conflict in parent-child relationship was negatively correlated with Home-based involvement (*r* = −0.11; *p* < 0.01); however, no significant correlations were found between Conflict in parent-child relationship and Home-school conferencing (*r* = −0.01; *p* > 0.05) or School-based involvement (*r* = −0.02, *p* > 0.05).

### Associations between family educational involvement, parent-child relationship, and social skills

Multivariate regression analysis revealed a similar pattern of the effects of family educational involvement and parent-child relationship on social skills among preschoolers. First, as shown in Table 3, the *R*^2^ value ranged from 0.31 to 0.37 for the seven SSIS-RS subscales, which suggests that over 30% of the variance in SSIS-RS subscale scores was predicted by the studied factors; according to Cohen (1988), this indicates a large effect.

**TABLE 3:** Standardized coefficients from multivariate regression analysis.

Coefficients	Communication		Cooperation		Assertion		Responsibility		Empathy		Engagement		Self-control	
	**β**	***SE***	**β**	***SE***	**β**	***SE***	**β**	***SE***	**β**	***SE***	**β**	***SE***	**β**	***SE***
SES	–0.01	0.01	–0.02	0.01	0.01	0.01	–0.02	0.01	0.00	0.01	–0.02	0.01	–0.01	0.01
AGE	0.07**	0.01	0.09**	0.01	0.06**	0.01	0.10**	0.01	0.07**	0.01	0.07**	0.01	0.09**	0.01
HSC	0.12**	0.02	0.10**	0.02	0.14**	0.02	0.11**	0.02	0.12**	0.02	0.12**	0.02	0.13**	0.02
SBI	–0.03	0.02	–0.03	0.03	–0.02	0.03	–0.02	0.03	–0.01	0.03	–0.01	0.02	–0.02	0.03
HBI	0.37**	0.02	0.36**	0.02	0.35**	0.02	0.35**	0.02	0.36**	0.02	0.34**	0.02	0.34**	0.02
Closeness	0.23**	0.02	0.20**	0.02	0.23**	0.02	0.19**	0.02	0.22**	0.02	0.22**	0.02	0.16**	0.02
Conflicts	−0.09**	0.02	−0.15**	0.02	−0.03*	0.02	−0.13**	0.02	−0.08**	0.02	−0.06**	0.02	−0.10**	0.02
Closeness ×HSC	0.00	0.03	0.01	0.03	0.00	0.03	0.02	0.03	0.02	0.03	0.00	0.03	0.02	0.03
Closeness ×SBI	0.02	0.03	0.02	0.03	0.03	0.03	0.01	0.03	0.01	0.03	0.02	0.03	0.02	0.03
Closeness ×HBI	0.07*	0.03	0.06*	0.03	0.06*	0.02	0.05*	0.03	0.06*	0.03	0.07*	0.03	0.05*	0.03
Conflicts ×HSC	0.05	0.03	0.06	0.04	0.03	0.03	0.06	0.04	0.04	0.04	0.04	0.03	0.05	0.03
Conflicts ×SBI	0.01	0.04	0.01	0.04	0.02	0.04	0.01	0.04	0.02	0.04	–0.01	0.04	0.04	0.04
Conflicts ×HBI	0.04	0.03	0.04	0.03	0.04	0.03	0.04	0.03	0.04	0.03	0.05	0.03	0.04	0.03
*R* ^2^	0.37		0.34		0.34		0.34		0.35		0.33		0.31	

HSC, home-school conferencing; SBI, school-based involvement; HBI, home-based involvement.

* 0.05; ** 0.01.

Second, of the three types of family educational involvement measured by FIQ-SF, significant main effects were detected for Home-school conferencing (*β_*Communication*_* = 0.12, *β_*Cooperation*_* = 0.10, *β_*Assertion*_* = 0.14, *β_*Responsibility*_* = 0.11, *β_*Empathy*_* = 0.12, *β_*Engagement*_* = 0.12, *β_*Self–Control*_* = 0.13; *p* < 0.01 for all effects) and Home-based involvement (*β_*Communication*_* = 0.37, *β_*Cooperation*_* = 0.36, *β_*Assertion*_* = 0.35, *β_*Responsibility*_* = 0.35, *β_*Empathy*_* = 0.36, *β_*Engagement*_* = 0.34, *β_*Self–Control*_* = 0.34; *p* < 0.01 for all effects). No statistically significant effects related to School-based involvement were detected.

Third, there were significant main effects of parent-child Closeness (*β_*Communication*_* = 0.23, *β_*Cooperation*_* = 0.20, *β_*Assertion*_* = 0.23, *β_*Responsibility*_* = 0.19, *β_*Empathy*_* = 0.22, *β_*Engagement*_* = 0.22, *β_*Self–Control*_* = 0.16; *p* < 0.01 for all effects) and Conflict (*β_*Communication*_* = −0.09, *β_*Cooperation*_* = −0.15, *β_*Assertion*_* = −0.03, *β_*Responsibility*_* = −0.13, *β_*Empathy*_* = −0.08, *β_*Engagement*_* = −0.06, *β_*Self–Control*_* = −0.10; *p* < 0.01 for all effects) on the scores of SSIS-RS subscales. Notably, across the SSIS-RS subscales, the impact of parent-child Conflict was relatively subtle compared to that of parent-child Closeness.

**FIGURE 2:**
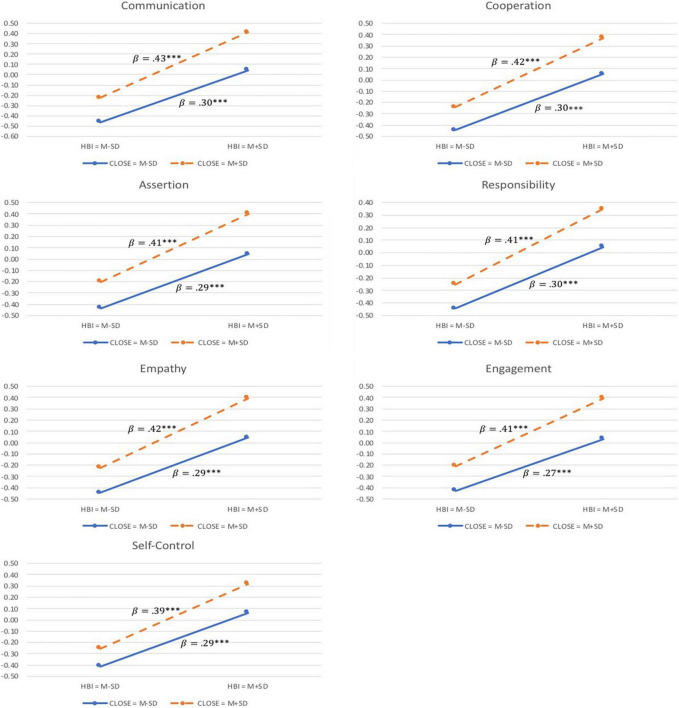
Moderation effect of parent-child closeness on the impact of home-based involvement on preschoolers’ social skills. βs are regression coefficients of Home-based Involvement from simple slope analyses. ***p < 0.001.

Lastly, of the six interactive effects between subscales of FIQ-SF and CPRS, only the interaction between parent-child Closeness and Home-based involvement was statistically significant, with standardized coefficient values ranging from 0.05 to 0.07 across the seven SSIS-RS subscales. This suggests that a close parent-child relationship positively moderates the effect of home-based involvement on social skills among preschoolers. Given the significant interaction of Closeness and Home-based involvement, we conducted two follow-up analyses at high (Mean + 1 SD) and low (Mean−1 SD) levels of Closeness, respectively. This simple slope analysis enables further examinations of the simple main effect of Home-based involvement on child social skills, and hence affords an in-depth understanding of the change in predictive effect of Home-based involvement as a function of Closeness. Accordingly, when Closeness was at low level, statistical significance was found on the simple main effect of Home-based involvement (*β_*Communication*_* = 0.30, *β_*Cooperation*_* = 0.30, *β_*Assertion*_* = 0.29, *β_*Responsibility*_* = 0.30, *β_*Empathy*_* = 0.29, *β_*Engagement*_* = 0.27, *β_*Self–Control*_* = 0.29; *p* < 0.001 for all effects). Similarly, for the high-level of Closeness, the simple main effect of Home-based Involvement was significant on all domains of child social skills (*β_*Communication*_* = 0.43, *β_*Cooperation*_* = 0.42, *β_*Assertion*_* = 0.41, *β_*Responsibility*_* = 0.41, *β_*Empathy*_* = 0.42, *β_*Engagement*_* = 0.41, *β_*Self–Control*_* = 0.39; *p* < 0.001 for all effects). This collectively suggested that a higher level of parental involvement at home was associated with a positive impact on children’s social skill development. Further, compared with children who had a less close relationship with their parents, Home-based involvement had a relatively stronger impact on social skills among children who maintained a close relationship with their parents. Visualizations of the interaction between Closeness and Home-based involvement are presented in Figure 2. Nevertheless, this interactive effect led to a subtle difference relative to the main effects of Home-based involvement and parent-child Closeness. Additionally, age showed significant yet subtle effects on SSIS-RS subscales, with standardized coefficient values varying from 0.06 to 0.10. No significant effect of family SES was found on any of the seven SSIS-RS subscales.

## Discussion

This study explored the effect of family educational involvement on preschoolers’ social skills and, of primary interest, the susceptibility of their associations to parent-child relationship (i.e., the moderator). As described, the scores for subscales of each measure (family educational involvement by FIQ-SF, social skills by SSIS-RS, and parent-child relationship by CPRS) are reported and were analyzed, affording a fine-grained investigation of their associations. In the sections that follow, we discuss the direct links between family educational involvement and preschoolers’ social skills, as well as the moderating role of parent-child relationship.

### Family educational involvement and preschoolers’ social skills

Our study found that Home-based involvement and Home-school conferencing, but not School-based involvement, were positively and significantly related to preschoolers’ social skills. Existing studies examining the impact of different dimensions of parental involvement have suggested a stronger relationship of home-based involvement with child outcomes relative to other types of involvement (Fantuzzo et al., 2004; Lau et al., 2011; Xia et al., 2020). Home-based involvement includes providing children with a stimulating home environment conducive to learning, such as offering learning materials, reading to children, discussing daily events, and creating learning experiences. During these activities, parents are modeling and reinforcing interpersonal interactions, as well as building relationships with their child; whereas, children can observe role models of appropriate behavior and practice social interaction skills with their parents. Children with highly involved parents in home settings tend to have increased ability to self-regulate (Mistry et al., 2010), higher self-esteem (Hung, 2005), better internal and external motivation (Mccollough and Ramirez, 2010), and more communication skills/interactions with peers and emotional regulation (Hill and Craft, 2003). Home-school conferencing involves the communication and interaction between parents and teachers about children’s educational experience and progress, such as talking about children’s difficulties or accomplishments. It provides parents (or teachers) with information regarding children’s learning and development at school (or at home), which is associated with higher levels of social skills (Powell et al., 2010). Home-school conferencing increases parents/teachers’ knowledge about the school/home’ expectations for behavior and strategies for improvement, and parents and teachers are likely to reach a consensus about appropriate behavior at both home and school (Hill and Taylor, 2004).

No significant influence of school-based involvement was found in this study. School-based involvement includes activities such as attending school events and volunteering in the classroom. Given that these activities entail direct parent contact with teachers and are less visible to children, previous research suggests that the impact of school-based involvement on child outcomes is limited (Fantuzzo et al., 2004; Dove et al., 2015). Furthermore, in the Chinese context, parents usually play a passive role in school-based involvement and lack opportunities to have constructive discussions with teachers about effective strategies to improve child outcomes (Xia et al., 2020). Although the positive effects of school-based involvement on the academic outcomes of children, such as reading and math, have been reported previously in studies based in Western culture (Schulting et al., 2005; Cooper et al., 2010), its impact on the social outcomes of children requires further investigation.

### The moderating role of parent-child relationship

Our findings indicated that parent-child closeness moderated the relationship between home-based involvement and preschoolers’ social skills. Specifically, home-based involvement and preschoolers’ social skills were more strongly positively associated when the parent-child relationship was characterized by high closeness compared with low closeness. This finding partially supports Darling and Steinberg (1993) Integrative Model of Parenting, which postulates that the emotional atmosphere of the parent-child relationship can change the effectiveness of parenting practices. The emotional climate provides a context from which parents practice their behaviors and interact with their children. In the context of a relationship with warmth and respect, children are more likely to engage in their parents’ socialization practices (Spera, 2005). Previously reported evidence also revealed a positive association between home-based involvement and child development outcomes when the parent and child have a close relationship (e.g., Simpkins et al., 2006; Stright and Yeo, 2014). When building a close relationship with their parents, children might perceive parental involvement activities at home as an expression of love, care, and emotional support; and thus were more motivated to learn, behaved more appropriately, and performed better (Xia et al., 2020). Increases in parent-child closeness may make it easier for children to internalize their parents’ views and actions through identification and modeling processes (Grolnick et al., 1997). In the context of this study, closeness in parent-child relationship may have helped children internalize the values of social interactions and facilitated their engagement in social behaviors (such as perspective-taking and self-control), leading to higher levels of social skills.

In this study, parent-child conflict was not identified as a significant moderator of the associations between family educational involvement and preschoolers’ social skills, consistent with findings from previous studies showing a lack of evidence for a significant moderating effect of parent-child conflict (e.g., Simpkins et al., 2006). Although the power of parental involvement may be reduced because of a poor quality parent-child relationship (Cooper et al., 2010), closeness and conflict in parent-child relationships contribute independently to the prediction of children’s social, emotional, and behavioral outcomes (Zhang, 2013). Theories of motivation place a great emphasis on the importance of positive relationships/interactions, rather than on the negative nature of relationships; this does not mean that negative aspects of a relationship, such as conflict, are not important, rather, parent-child conflict may have a significant impact when the level of negativity or conflict is relatively high. Notably, participants in this study reported a relatively low level of parent-child conflict. Further research involving samples of Chinese parents with high levels of parent-child conflict is needed to examine whether parent-child conflict moderates the association between parental involvement and child development outcomes.

### Implications and limitations

This study is among the first to explore the moderating effect of parent-child relationship on the association between family educational involvement and preschoolers’ social skills in the Chinese context. This study has examined the mechanisms underlying how family educational involvement is moderated by parent-child relationship and related to preschoolers’ social skills in the Chinese context. Further, the investigation extends our understanding of family processes and provides empirical evidence of the importance of family educational involvement and parent-child relationship in facilitating the development of social skills in preschoolers. As families are considered as a key source of support for young children, promoting family educational involvement is a growing priority for early childhood policymakers and practitioners. In this study, we found that home-based involvement had the strongest evidence for an association with children’s social skills, indicating that increasing home-based parental involvement may better promote children’s social skills. That is, improving parental involvement in the home environment has the potential to be an effective way to support the social skills of preschoolers. Furthermore, findings from this study underscore the significance of considering the joint influences of family educational involvement and parent-child relationship. Specifically, the relations between home-based involvement and preschoolers’ social skills were enhanced when parents and their child demonstrate high levels of closeness. Implications for practice are highlighted with a particular emphasis on strengthening the home-based involvement and parent-child closeness. Educators should advise parents to increase their involvement in the home setting, while maintaining a close relationship with their children. Both the quantity and quality dimensions of family educational involvement are important. The amount of family educational involvement does not necessarily produce benefits, while the quality of family educational involvement may exert a greater influence on child development outcomes (Moroni et al., 2015). It is necessary to consider the emotional climate that parents establish by participating in their children’s learning and development. Educators should convey to parents the significance of family educational involvement in a close and warm atmosphere, and suggest ways to implement high-quality home-based involvement in their children’s learning and development.

A number of limitations of the present study are noted, which could be further improved in future studies. First, although a relatively large sample was involved, participants were only recruited from one province in China, which potentially limits the generalizability of our findings to other regions of China. Future studies are needed to involve participants from more diverse economic, geographic, and educational backgrounds. Second, this study collected data from parents and children during a single period of time. Such a cross-sectional design enables examination of concurrent relationships, but provides little information regarding the long-term impact of family involvement on the development of social skills of young children, or on the moderating role of parent-child relationship over time. Longitudinal studies are needed to gain a more thorough understanding of the effects of family involvement and parent-child relationship. Third, parental involvement and parent-child relationship were measured *via* self-report, and preschoolers’ social skills were parent-reported. Future studies employing other measures, such as independent observation and direct child assessment, will provide a deeper understanding of the relationships among the studied variables. Fourth, the respondents in this study were children’s primary caregivers, and we did not distinguish fathers and mothers in terms of their involvement and relationships with their children. The level of parental involvement and the pattern of parent-child relationship may differ between fathers and mothers. Future research may compare the influence of fathers’ and mothers’ involvement/relationship on their children’s early learning and development, as well as investigating their underlying mechanisms.

## Conclusion

This study confirms the benefits of family educational involvement in child outcomes. Among different dimensions of family educational involvement, home-based involvement evidenced the strongest relationship to preschoolers’ social skills. This study contributes to the existing literature by empirically investigating the moderating role of parent-child relationship in the associations between family educational involvement and preschoolers’ social skills. According to our findings, home-based involvement is more beneficial for children when the parent and child share a relationship characterized by high levels of closeness than for those in a relationship with low closeness. These findings reinforce the importance of considering home-based involvement in the context of parent-child closeness in fostering preschoolers’ social skills.

## Data availability statement

The raw data supporting the conclusions of this article will be made available by the authors, without undue reservation.

## Ethics statement

The studies involving human participants were reviewed and approved by the Capital Normal University. Written informed consent for participation was not required for this study in accordance with the national legislation and the institutional requirements.

## Author contributions

Conceptualization and design of the research were conducted by HL, YQ, and LL. All authors contributed to the data analyses and interpretation, and were involved in the manuscript writing and revisions.

## References

[B1] Boyer B. P. Scott J. K. Nelson J. A. (2016). Maternal conflict behavior profiles and child social skills. *Soc. Dev.* 25 759–776. 10.1111/sode.12169

[B2] Brajša-Žganec A. Merkaš M. Šakić Veliæ M. (2019). The relations of parental supervision, parental school involvement, and child’s social competence with school achievement in primary school. *Psychol. Sch.* 56 1246–1258. 10.1002/pits.22273

[B3] Bulotsky-Shearer R. J. Bouza J. Bichay K. Fernandez V. A. Gaona Hernandez P. (2016). Extending the validity of the family involvement questionnaire-short form for culturally and linguistically diverse families from low-income backgrounds. *Psychol. Sch.* 35 911–925. 10.1002/pits.21953

[B4] Cheung C. S. Pomerantz E. M. (2011). Parents’ involvement in children’s learning in the United States and China: implications for children’s academic and emotional adjustment. *Child Dev.* 82 932–950. 10.1111/j.1467-8624.2011.01582.x 21418057 PMC3089668

[B5] Cohen F. Anders Y. (2020). Family involvement in early childhood education and care and its effects on the social-emotional and language skills of 3-year-old children. *Sch. Eff. Sch. Improv.* 31 125–142. 10.1080/09243453.2019.1646293

[B6] Cohen J. (1988). *Statistical Power Analysis for the Behavioral Sciences*, 2nd Edn. Hillsdale, NJ: Lawrence Erlbaum Associates.

[B7] Connell C. M. Prinz R. J. (2002). The impact of childcare and parent-child interactions on school readiness and social skills development for low-income African American children. *J. Sch. Psychol.* 40 177–193. 10.1016/S0022-4405(02)00090-0

[B8] Cooper C. E. Crosnoe R. Suizzo M.-A. Pituch K. A. (2010). Poverty, race, and parental involvement during the transition to elementary school. *J. Fam. Issues* 31 859–883. 10.1177/0192513X09351515

[B9] Darling N. Steinberg L. (1993). Parenting style as context: an integrative model. *Psychol. Bull.* 113 487–496. 10.1037/0033-2909.113.3.487

[B10] Denham S. A. (2007). Dealing with feelings: how children negotiate the worlds of emotions and social relationships. *Cogn. Creier Comport.* 11 1–48.

[B11] Denham S. A. Mitchell-Copeland J. Strandberg K. Auerbach S. Blair K. (1997). Parental contributions to preschoolers’ emotional competence: direct and indirect effects. *Motiv. Emot.* 21 65–86. 10.1023/A:1024426431247

[B12] Dove M. K. Neuharth-Pritchett S. Wright D. W. Wallinga C. (2015). Parental involvement routines and Former Head Start children’s literacy outcomes. *J. Res. Child. Educ.* 29, 173–186. 10.1080/02568543.2015.1011360

[B13] Edwards C. P. Sheridan S. M. Knoche L. (2010). “Parent-child relationships in early learning,” in *International Encyclopedia of Education*, eds Baker E. Peterson P. McGaw B. (Amsterdam: Elsevier), 438–443.

[B14] El Nokali N. E. Bachman H. J. Votruba-Drzal E. (2010). Parent involvement and children’s academic and social development in elementary school. *Child Dev.* 81 988–1005. 10.1111/j.1467-8624.2010.01447.x 20573118 PMC2973328

[B15] Epstein J. L. (1995). School/family/community partnerships: caring for the children we share. *Phi Delta Kappan.* 76 701–712. 10.1177/003172171009200326

[B16] Fan X. Chen M. (2001). Parental involvement and students’ academic achievement: a meta-analysis. *Educ. Psychol. Rev.* 13 1–22. 10.1023/A:1009048817385

[B17] Fantuzzo J. Gadsden V. Li F. Sproul F. McDermott P. Hightower D. (2013). Multiple dimensions of family engagement in early childhood education: evidence for a short form of the family involvement questionnaire. *Early Child. Res. Q.* 28 734–742. 10.1016/j.ecresq.2013.07.001

[B18] Fantuzzo J. McWayne C. Perry M. A. Childs S. (2004). Multiple dimensions of family involvement and their relations to behavioral and learning competencies for urban, low-income children. *School Psych. Rev.* 33 467–480. 10.1080/02796015.2004.12086262

[B19] Fantuzzo J. Tighe E. Childs S. (2000). Family involvement questionnaire: a multivariate assessment of family participation in early childhood education. *J. Educ. Psychol.* 92 367–376. 10.1037/0022-0663.92.2.367

[B20] Gresham F. M. Elliott S. N. (2008). *Social Skills Improvement System—Rating Scales.* Minneapolis, MN: Pearson Assessments.

[B21] Grolnick W. S. Benjet C. Kurowski C. O. Apostoleris N. H. (1997). Predictors of parent involvement in children’s schooling. *J. Educ. Psychol*. 89, 538–548. 10.1037/0022-0663.89.3.538

[B22] Hill N. E. Craft S. A. (2003). Parent-school involvement and school performance: mediated pathways among socioeconomically comparable African American and Euro-American families. *J. Educ. Psychol.* 95 74–83. 10.1037/0022-0663.95.1.74

[B23] Hill N. E. Taylor L. C. (2004). Parental school involvement and children’s academic achievement: pragmatics and issues. *Curr. Dir. Psychol. Sci.* 13 161–164. 10.1111/j.0963-7214.2004.00298.x

[B24] Hu B. Y. Fan X. Wu Z. LoCasale-Crouch J. Yang N. Zhang J. (2017). Teacher-child interactions and children’s cognitive and social skills in Chinese preschool classrooms. *Child. Youth Serv. Rev.* 79 78–86. 10.1016/j.childyouth.2017.05.028

[B25] Hung C.-L. (2005). Family background, parental involvement and environmental influences on Taiwanese children. *Alberta J. Educ. Res.* 51 261–276. 10.1111/j.1365-2214.2008.00898.x 18991969

[B26] Kang J. Horn E. M. Palmer S. (2017). Influences of family involvement in kindergarten transition activities on children’s early school adjustment. *Early Child. Educ. J.* 45 789–800. 10.1007/s10643-016-0828-4

[B27] Kline R. B. (2016). *Principles and Practice of Structural Equation Modeling*, 4th Edn. New York, NY: Guilford Press.

[B28] Lai K. Green S. B. (2016). The problem with having two watches: assessment of fit when RMSEA and CFI disagree. *Multivariate Behav. Res.* 51 220–239. 10.1080/00273171.2015.1134306 27014948

[B29] Laible D. J. Thompson R. A. (2002). Mother-child conflict in the toddler years: lessons in emotion, morality, and relationships. *Child Dev.* 73 1187–1203. 10.1111/1467-8624.00466 12146742

[B30] Lau E. Y. H. Li H. Rao N. (2011). Parental involvement and children’s readiness for school in China. *Educ. Res.* 53 95–113. 10.1080/00131881.2011.552243

[B31] Leidy M. S. Guerra N. G. Toro R. I. (2010). Positive parenting, family cohesion, and child social competence among immigrant Latino families. *J. Fam. Psychol.* 24 252–260. 10.1037/a0019407 20545398

[B32] Lin N. Bian Y. (1991). Getting ahead in urban China. *Am. J. Soc.* 97 657–688. 10.1086/229816

[B33] Liu Q. Li X. (2019). Revision of family involvement questionnaire-short form in parents of preschool children in China. *Chin. J. Clin. Psychol.* 27 49–53.

[B34] McClelland M. M. Morrison F. J. Holmes D. L. (2000). Children at risk for early academic problems: the role of learning related social skills. *Early Child. Res. Q.* 15 307–329. 10.1016/S0885-2006(00)00069-7

[B35] Mccollough C. Ramirez O. (2010). Connecting math and science to home, school and community through preservice teacher education. *Acad. Leadersh.* 8 1–11.

[B36] McWayne C. M. Manz P. H. Ginsburg-Block M. D. (2015). Examination of the Family Involvement Questionnaire-Early Childhood (FIQ-EC) with low-income. Latino families of young children. *Int. J. Educ. Psychol.* 3 117–134. 10.1080/21683603.2014.950439

[B37] McWayne C. Hampton V. Fantuzzo J. Cohen H. L. Sekino Y. (2004). A multivariate examination of parent involvement and the social and academic competencies of urban kindergarten children. *Psychol. Sch.* 41 363–377. 10.1002/pits.10163

[B38] Mistry R. S. Benner A. D. Biesanz J. C. Clark S. L. Howes C. (2010). Family and social risk, and parental investments during the early childhood years as predictors of low-income children’s school readiness outcomes. *Early Child. Res. Q.* 25 432–449. 10.1016/j.ecresq.2010.01.002

[B39] Moroni S. Dumont H. Trautwein U. Niggli A. Baeriswyl F. (2015). The need to distinguish between quantity and quality in research on parental involvement: the example of parental help with homework. *J. Educ. Res.* 108 417–431. 10.1080/00220671.2014.901283

[B40] Muthén L. K. Muthén B. (1998–2017). *Mplus User’s Guide.* Los Angeles, CA: Muthén & Muthén.

[B41] National Bureau of Statistics of China (2021). *National Data.* Available Online at: https://data.stats.gov.cn/english/easyquery.htm?cn=E0102 (accessed January 30, 2022).

[B42] Nelson J. A. (2015). Child reactivity moderates the over-time association between mother–child conflict quality and externalizing problems. *Int. J. Behav. Dev.* 39 376–382. 10.1177/0165025415573643

[B43] Pang I. W. (2004). School-family community partnership in Hong Kong: perspectives and challenges. *Educ. Res. Policy Pract.* 3 109–125. 10.1007/s10671-004-5556-7

[B44] Pianta R. C. (1992). *Child-Parent Relationship Scale.* Available Online at: https://curry.virginia.edu/faculty-research/centers-labs-projects/castl/measures-developed-robert-c-pianta-phd (accessed January 30, 2022).

[B45] Pomerantz E. M. Moorman E. A. Litwack S. D. (2007). The how, whom, and why of parents’ involvement in children’s academic lives: more is not always better. *Rev. Educ. Res.* 77 373–410. 10.3102/003465430305567

[B46] Powell D. R. Son S.-H. File N. San Juan R. R. (2010). Parent–school relationships and children’s academic and social outcomes in public school pre-kindergarten. *J. Sch. Psychol.* 48 269–292. 10.1016/j.jsp.2010.03.002 20609850

[B47] Rhoades B. L. Warren H. K. Domitrovich C. E. Greenberg M. T. (2011). Examining the link between preschool social-emotional competence and first grade academic achievement: the role of attention skills. *Early Child. Res. Q.* 26 182–191. 10.1016/j.ecresq.2010.07.003

[B48] Rimm-Kaufman S. E. Pianta R. C. Cox M. J. Bradley R. H. (2003). Teacher-rated family involvement and children’s social and academic outcomes in kindergarten. *Early Educ. Dev.* 14 179–198. 10.1207/s15566935eed1402_3

[B49] Schulting A. B. Malone P. S. Dodge K. A. (2005). The effect of school-based kindergarten transition policies and practices on child academic outcomes. *Dev. Psychol.* 41 860–871. 10.1037/0012-1649.41.6.860 16351333 PMC2757260

[B50] See B. H. Gorard S. (2015). The role of parents in young people’s education—A critical review of the causal evidence. *Oxf. Rev. Educ.* 41 346–366. 10.1080/03054985.2015.1031648

[B51] Seginer R. (2006). Parents’ educational involvement: a developmental ecology perspective. *Parent. Sci. Pract.* 6 1–48. 10.1207/s15327922par0601_1

[B52] Simpkins S. D. Weiss H. B. McCartney K. Kreider H. M. Dearing E. (2006). Mother-child relationship as a moderator of the relation between family educational involvement and child achievement. *Parent. Sci. Pract.* 6 49–57. 10.1207/s15327922par0601_2

[B53] Spera C. (2005). A review of the relationship among parenting practices, parenting styles, and adolescent school achievement. *Educ. Psychol. Rev.* 17 125–146. 10.1007/s10648-005-3950-1

[B54] Stevens J. (1996). *Applied Multivariate Statistics for the Social Sciences.* Mahwah, NJ: Lawrence Erlbaum Associates.

[B55] Stright A. D. Yeo K. L. (2014). Maternal parenting styles, school involvement, and children’s school achievement and conduct in Singapore. *J. Educ. Psychol.* 106 301–314. 10.1037/a0033821

[B56] Torres N. Veríssimo M. Monteiro L. Ribeiro O. Santos A. J. (2014). Domains of father involvement, social competence and problem behavior in preschool children. *J. Fam. Stud.* 20 188–203. 10.1080/13229400.2014.11082006

[B57] Weaver C. M. Shaw D. S. Crossan J. L. Dishion T. J. Wilson M. N. (2015). Parent–child conflict and early childhood adjustment in two-parent low-income families: parallel developmental processes. *Child Psychiatry Hum. Dev.* 46 94–107. 10.1007/s10578-014-0455-5 24610382 PMC4523123

[B58] Wu Z. Mak M. C. K. Hu B. Y. He J. Fan X. (2019). A validation of the social skills domain of the social skills improvement system-rating scales with Chinese preschoolers. *Psychol. Sch.* 56 126–147. 10.1002/pits.22193

[B59] Xia X. Hackett R. K. Webster L. (2020). Chinese parental involvement and children’s school readiness: the moderating role of parenting style. *Early Educ. Dev.* 31 250–268. 10.1080/10409289.2019.1643439

[B60] Xu L. Liu L. Li Y. Liu L. Huntsinger C. S. (2018). Parent–child relationships and Chinese children’s social adaptations: gender difference in parent–child dyads. *Pers. Relatsh.* 25 462–479. 10.1111/pere.12254

[B61] Zhang X. (2013). Chinese children’s relationships with mothers during the transition to nursery care: changes and associations with later growth in social competence. *Infant. Ment. Health J.* 34 60–71. 10.1002/imhj.21354

[B62] Zhang X. Chen H. (2010). Reciprocal influences between parents’ perceptions of mother-child and father-child relationships: a short-term longitudinal study in Chinese preschoolers. *J. Genet. Psychol.* 171 22–34. 10.1080/00221320903300387 20333893

[B63] Zhang X. Chen H. Zhang G. (2008). Children’s relationships with mothers and teachers: linkages to problem behavior in their first preschool years. *Acta Psychol. Sin.* 40 418–426.

[B64] Zhou Q. Eisenberg N. Losoya S. H. Fabes R. A. Reiser M. Guthrie I. K. (2002). The relations of parental warmth and positive expressiveness to children’s empathy-related responding and social functioning: a longitudinal study. *Child Dev.* 73 893–915. 10.1111/1467-8624.00446 12038559

