# Supplementary material for: Exploring family educational involvement and social skills in Chinese preschoolers: The moderating role of parent-child relationship
Source: Front Psychol. 2022 Aug 1;13:911421. doi: 10.3389/fpsyg.2022.911421 (PMC9376233; doi:10.3389/fpsyg.2022.911421)
Supplement: Supplementary file 1 [file Table_1.DOCX]

Exploring Family Educational Involvement and Social Skills in Chinese Preschoolers: The Moderating Role of Parent-Child Relationship

Supplementary Material

**Supplementary Table 1.** Summary of CFA Model Fit

| Measure | $\chi^{2}$ | *df* | *p* | RMSEA | 90%*CI* | CFI/TLI | SRMR |
| --- | --- | --- | --- | --- | --- | --- | --- |
| SSIS-RS | 13785.06 | 968 | .00 | .052 | [.051, .053] | .95/.95 | .05 |
| FIQ-SF | 8709.53 | 186 | .00 | .096 | [.095, .098] | .99/.98 | .04 |
| CPRS | 2826.31 | 89 | .00 | .079 | [.076, .081] | .96/.95 | .06 |

*Note:* SSIS-RS = Social Skills Improvement System-Rating Scales; FIQ-SF = Family Involvement Questionnaire-Short Form; CPRS = Child-Parent Relationship Scale.

RMSEA = Root Mean Square Error of Approximation; CFI = Comparative Fit Index; TLI = Tucker-Lewis Index; SRMR = Standardized Root Mean Square Residual.

**Supplementary Table 2.** Standardized Factor Loadings from CFA for SSIS-RS

| Communication | | | Cooperation | | | Assertion | | | Responsibility | | | Empathy | | | Engagement | | | Self-Control | | |
| --- | --- | --- | --- | --- | --- | --- | --- | --- | --- | --- | --- | --- | --- | --- | --- | --- | --- | --- | --- | --- |
| Item | $\lambda$ | SE | Item | $\lambda$ | SE | Item | $\lambda$ | SE | Item | $\lambda$ | SE | Item | $\lambda$ | SE | Item | $\lambda$ | SE | Item | $\lambda$ | SE |
| S4 | 0.58 | 0.01 | S9 | 0.80 | 0.01 | S7 | 0.61 | 0.01 | S6 | 0.63 | 0.01 | S51 | 0.80 | 0.01 | S10 | 0.77 | 0.01 | S28 | 0.85 | 0.01 |
| S2 | 0.65 | 0.01 | S1 | 0.64 | 0.01 | S23 | 0.59 | 0.01 | S13 | 0.67 | 0.01 | S49 | 0.67 | 0.01 | S30 | 0.82 | 0.01 | S20 | 0.56 | 0.01 |
| S14 | 0.75 | 0.01 | S25 | 0.70 | 0.01 | S11 | 0.79 | 0.01 | S40 | 0.75 | 0.01 | S5 | 0.66 | 0.01 | S16 | 0.81 | 0.01 | S32 | 0.69 | 0.01 |
| S52 | 0.64 | 0.01 | S17 | 0.74 | 0.01 | S36 | 0.72 | 0.01 | S29 | 0.87 | 0.01 | S38 | 0.84 | 0.01 | S8 | 0.79 | 0.01 | S18 | 0.61 | 0.01 |
| S22 | 0.62 | 0.01 | S26 | 0.77 | 0.01 | S31 | 0.70 | 0.01 | S24 | 0.76 | 0.01 | S34 | 0.77 | 0.01 | S46 | 0.70 | 0.01 | S39 | 0.56 | 0.01 |
| S12 | 0.79 | 0.01 | S50 | 0.73 | 0.01 | S53 | 0.59 | 0.01 | S21 | 0.77 | 0.01 | S15 | 0.83 | 0.01 | S37 | 0.80 | 0.01 | S42 | 0.65 | 0.01 |
| S43 | 0.63 | 0.01 |  |  |  | S27 | 0.72 | 0.01 |  |  |  |  |  |  | S41 | 0.74 | 0.01 | S44 | 0.76 | 0.01 |

*Note.* FIQ-SF = Family Involvement Questionnaire-Short Form.

**Supplementary Table 3.** Standardized Factor Loadings from CFA for FIQ-SF

| Home-school Conferencing | | |  | School-based Involvement | | |  | Home-based involvement | | |
| --- | --- | --- | --- | --- | --- | --- | --- | --- | --- | --- |
| Item | $\lambda$ | *SE* |  | Item | $\lambda$ | *SE* |  | Item | $\lambda$ | *SE* |
| F1 | 0.854 | 0.004 |  | F8 | 0.863 | 0.004 |  | F15 | 0.811 | 0.006 |
| F2 | 0.951 | 0.001 |  | F9 | 0.907 | 0.003 |  | F16 | 0.793 | 0.007 |
| F3 | 0.976 | 0.001 |  | F10 | 0.916 | 0.003 |  | F17 | 0.846 | 0.005 |
| F4 | 0.951 | 0.001 |  | F11 | 0.856 | 0.006 |  | F18 | 0.842 | 0.005 |
| F5 | 0.942 | 0.002 |  | F12 | 0.892 | 0.004 |  | F19 | 0.883 | 0.003 |
| F6 | 0.933 | 0.002 |  | F13 | 0.892 | 0.003 |  | F20 | 0.866 | 0.004 |
| F7 | 0.923 | 0.002 |  | F14 | 0.849 | 0.005 |  | F21 | 0.917 | 0.003 |
| *Note.* SSIS-RS = Social Skills Improvement System-Rating Scales. | | | | | | | | | | |

**Supplementary Table 4.** Standardized Factor Loadings from CFA for CPRS

| Closeness | | |  | Conflict | | |
| --- | --- | --- | --- | --- | --- | --- |
| Item | $\lambda$ | *SE* |  | Item | $\lambda$ | *SE* |
| R1 | 0.749 | 0.01 |  | R2 | 0.639 | 0.01 |
| R3 | 0.763 | 0.01 |  | R4 | 0.699 | 0.01 |
| R5 | 0.881 | 0.01 |  | R8 | 0.642 | 0.01 |
| R6 | 0.908 | 0.01 |  | R10 | 0.721 | 0.01 |
| R7 | 0.834 | 0.01 |  | R11 | 0.637 | 0.01 |
| R9 | 0.588 | 0.01 |  | R12 | 0.613 | 0.01 |
| R15 | 0.759 | 0.01 |  | R13 | 0.734 | 0.01 |
|  |  |  |  | R14 | 0.824 | 0.01 |

*Note.* CPRS = Child-Parent Relationship Scale.
